# Continental shelves as potential resource of rare earth elements

**DOI:** 10.1038/s41598-017-06380-z

**Published:** 2017-07-19

**Authors:** Olivier Pourret, Johann Tuduri

**Affiliations:** 1 UniLaSalle, 19 rue Pierre Waguet, 60026 Beauvais cedex, France; 2 BRGM, 3 avenue Claude Guillemin, 45100 Orléans, France

## Abstract

The results of this study allow the reassessment of the rare earth elements (REE) external cycle. Indeed, the river input to the oceans has relatively flat REE patterns without cerium (Ce) anomalies, whereas oceanic REE patterns exhibit strong negative Ce anomalies and heavy REE enrichment. Indeed, the processes at the origin of seawater REE patterns are commonly thought to occur within the ocean masses themselves. However, the results from the present study illustrate that seawater-like REE patterns already occur in the truly dissolved pool of river input. This leads us to favor a partial or complete removal of the colloidal REE pool during estuarine mixing by coagulation, as previously shown for dissolved humic acids and iron. In this latter case, REE fractionation occurs because colloidal and truly dissolved pools have different REE patterns. Thus, the REE patterns of seawater could be the combination of both intra-oceanic and riverine processes. In this study, we show that the Atlantic continental shelves could be considered potential REE traps, suggesting further that shelf sediments could potentially become a resource for REE, similar to metalliferous deep sea sediments.

## Introduction

Rare earth elements (REE) have received considerable attention in the past ten years, in part as a result of the tightening of export quotas from the monopolistic Chinese producers which has recently sparked a wave of speculation on REE prices, as observed in 2011. Nowadays, REE are still considered critical because they are a key component of the transition towards a competitive and low-carbon energy economy^1–3^. Thus they are essential in a wide variety of applications such as direct drive wind turbines, electric and hybrid vehicles, low-energy lighting^4, 5^. With a demand for REE thought to be growing at a rate of approximately 5–10% per year^6^, alternative primary and secondary REE supply sources must be found. However, even if recycling of scrap consumer electronics and technical industrial components will increasingly contribute to the REE supply in the near future^7^, it will not be able to meet the increasing demand^8^. Thus mining of natural primary deposits is expected to remain as the major source of REE^9^. However, efforts must be made on the exploration of new REE enriched deposits with low environmental impact. In this respect, high concentrations of REE have been reported from deep-ocean manganese nodules^10, 11^, iron-manganese crusts^10, 12^ and deep-sea muds of the Pacific Ocean floor^13^, which are being studied in detail for their economic potential. Oceans are often considered as being in a chemical steady state, thus displaying an elemental balance maintained by input/output rates. River inputs are well constrained^14^ and the behavior of REE during mixing of river and sea water is well studied^15–17^. However, as discussed by Rasmussen *et al*.^18^ and Lacan & Jeandel^19^ the amount of REE delivered to the coastal shelves and oceans must be significantly larger than previously estimated. Indeed the total burial flux of light REE (see below) is about 50 times greater than previously determined for deep-sea sediments^18^. Recently, (i) Freslon *et al*.^20^ highlight that sedimentary organic matter displays high REE concentrations and (ii) Rousseau *et al*.^21^ show that the dissolved REE speciation across the Amazon estuary salinity gradient is mainly controlled by coarse colloidal organic fraction which is progressively removed by coagulation as previously evidenced by Sholkovitz *et al*.^22^. In this context, the REE organic pool should be further considered. We report here a potential resource for REE with a focus on Atlantic continental shelves.

## Methods

Historically, the REE have been used to trace mass transfers during geological processes occurring both in the Earth’s interior and on the surface^23^. As a matter of fact, these elements have been typically divided into light REE (LREE; La-Nd), middle REE (MREE; Sm-Tb) and heavy REE (HREE; Dy-Lu) due to their contrasting geochemical behaviour. Their extremely low concentrations in nature imply that they can passively record mass transfers with little perturbation from thermodynamic interactions^23^. For this reason, we consider below that the transfer of stream water to the oceans leads to estuaries where mixtures of organic and inorganic phases are separated. Thus, about 90% of REE are removed from the solution by flocculation of colloidal material (from 86% for Lu to 95% for La)^24^. Inorganic REE remaining in solution can be also sorbed and thus removed from solution when coming into contact with highly reactive particles (such as Fe-Mn oxyhydroxides)^14, 25^, however this proportion is minimal (only 1% of REE compared to a withdrawal of 90% by colloidal flocculation). Thus, the REE patterns of the oceans appear to reflect those of the inorganic river water fraction^26^. Such information is therefore unknown, and only apparent when the inorganic and colloidal organic phases are separated, for example, during estuarine mixing. The low-temperature aqueous REE behavior was investigated by focusing on available dissolved REE concentration data in selected studies on the (i) Dordogne^27^ and Garonne rivers then Gironde estuary (ii) Congo river^28^ and its estuary (unpublished results from Germain Bayon), (iii) Amazon river^29^ and estuary^21^, (iv) sedimentary organic matters^20^ and (v) Ordovician organic rich grey monazites^30^. The Amazon and Congo rivers were selected as they are the two majors systems flowing into the Atlantic Ocean. The Dordogne river was chosen to highlight the behavior of a smaller river system for which the estuarine behavior has been extensively investigated in the Gironde estuary (France)^31^. It must be noted that the Gironde estuary corresponds to the confluence of the Garonne and Dordogne rivers in the Atlantic Ocean.

## Results and Discussion

**Figure 1:**
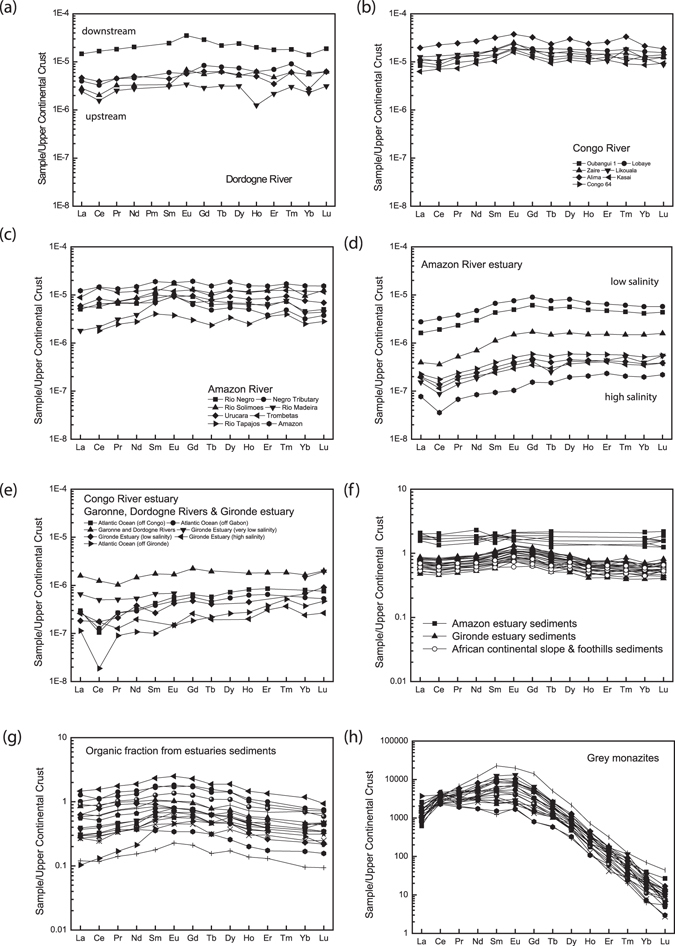
Upper continental crust (UCC)-normalized REE patterns in samples from (**a**) Dordogne River basin^27^, (**b**) Congo River basin^28^, (**c**) Amazon River basin^29^, (**d**) Amazon River estuary^21^, (**e**) Congo River estuary (unpublished results from Germain Bayon) and Dordogne, Garonne Rivers and Gironde estuary^31^, (**f**) African continental slope and foothill sediments^33^, Amazon estuary sediments^34^ and Gironde estuary sediments^35^, (**g**) organic fraction from estuaries sediments^20^ and (**h**) Ordovician organic rich grey monazites (each sample corresponds to a different monazite)^30^. UCC values are from McLennan^36^.

For the three studied rivers flowing into the Atlantic Ocean (i.e., Amazon, Congo and Dordogne rivers; Fig. 1), it can be observed that the REE patterns of upstream samples (Fig. 1a) depict low REE concentration and a negative cerium (Ce) anomaly, whereas more organic samples downstream (Fig. 1a,b,c) display higher REE concentrations (>one order of magnitude) and MREE enriched patterns with no Ce anomaly. This corresponds to an organic sedimentary input (with for example up to 15–20 mg/L of dissolved organic carbon in the Dordogne river)^27^. It can also be observed that in the Amazon and Gironde estuaries (Fig. 1d,e), the REE become progressively less concentrated (up 2–3 orders of magnitude) as the salinity increases. In addition, MREE patterns evolve to more HREE enriched patterns with a gradual development of a negative Ce anomaly. Similar MREE patterns are also found in the different estuary sediments (Fig. 1f) and in the organic fractions from sediments of these different estuaries (Fig. 1g) and are further observed in organic rich grey monazites (Fig. 1h), as previously observed on humic substances^32^.

**Figure 2:**
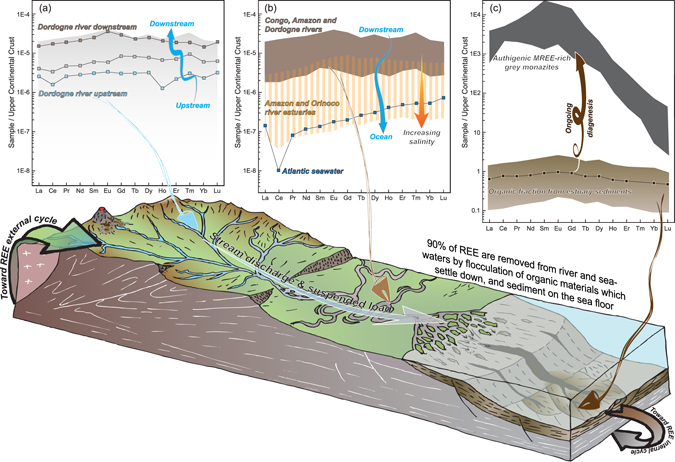
Sketch illustrating the REE external cycle and summarizing the processes responsible for REE fractionation from river water to organic sediments. UCC-normalized REE patterns are detailed in Fig. 1, and Atlantic seawater is from Freslon *et al*.^20^. UCC-normalized patterns for (**a**) river water, (**b**) river, estuarine and seawater, and (**c**) sedimentary organic matter and authigenic monazites.

Results highlighted in this short communication thus allow reassessing the REE external cycle (Fig. 2). The river input to the oceans has relatively flat REE patterns without a Ce anomaly, whereas oceanic REE patterns exhibit strong negative Ce anomalies and HREE enrichment. Indeed, the processes at the origin of seawater REE patterns are commonly thought to occur within the ocean masses themselves. However, the results highlighted in this short communication show that seawater-like REE patterns originate in the dissolved pool of river input (i.e., uphill watershed). Therefore, a partial or complete removal of the colloidal REE pool during estuarine mixing by coagulation is expected, as previously shown for dissolved organic matter (DOM) and iron^22^. Indeed, Rousseau *et al*.^21^ and Osborne *et al*.^37^ illustrated that both Amazon and Orinoco Rivers REE patterns lose their typical MREE enrichments. By performing ultrafiltration experiments, Rousseau *et al*.^21^ evidenced a LREE enrichment in the coarse colloidal fraction compared with the fine colloidal fraction and the truly dissolved phase. Humic substances as well as Fe-Mn oxyhydroxides (corresponding to the colloidal fraction) will favor a MREE enrichment whereas carbonate ligands (corresponding to the major anions in the truly dissolved phase) will enhanced HREE enrichment. They associate this fractionation to colloid coagulation. These MREE patterns gradually evolves towards HREE-enriched patterns that are more similar to Atlantic Ocean water^17^. REE fractionation occurs because colloidal and truly dissolved pools have different REE patterns (i.e., MREE shape-bell pattern vs HREE enriched pattern). Thus, REE patterns of seawater could be the combination of both intra-oceanic and riverine processes.

Merschel *et al*.^38^ show that estuarine processes may affect the flux of DOM, Fe and REE into the ocean via salt-induced coagulation and subsequent removal of river-borne nano-particles and colloids. In the near shore environment, DOM, Fe and REE are removed, at different rates, along the increasing salinity gradient of estuaries and shelves^14, 21, 37^. The seawater REE signature could be thus inherited from river water^39^. Commonly, REE signatures of ocean water are usually considered to reflect (i) the respective REE inputs from rivers, aeolian transport, hydrothermal vents and dissolution of marine carbonates and (ii) interactions with the biogeochemical cycles, involving REE-removal from surface waters by adsorption onto settling Fe-Mn particles. The strong Ce depletion and the HREE-enrichment of ocean waters are commonly attributed to the redox chemistry of Ce and to the high stability constants of HREE carbonate complexes^14, 25^. Nevertheless, different processes may lead to REE and/or Ce removal from solution. The most often discussed hypothesis is the occurrence of REE fractionation during estuarine mixing, enhanced by sorption on particles with extremely high surface reactivity rather than active microbial uptake, yet the exact nature of these particles (e.g., containing hydrous manganese oxide) is uncertain^40^.

In this context, the Atlantic continental shelves could be considered potential REE traps and shelf sediments would, similar to metalliferous deep sea sediments^13^, represent a potential REE resource. This latter hypothesis is illustrated by recent results from Freslon *et al*.^20^ and Rousseau *et al*.^21^. Indeed, by analyzing various fractions (detrital, Fe-Mn oxides, organic compounds) of sediments deposited in river estuaries, they proposed that organic matter is a major REE scavenger and possibly plays an important role in the oceanic REE budget (i.e., through direct scavenging and remineralization within the water column, up to 14% for the Congo Basin)^20^. Although high REE contents may be found in selected organic components (up to 350 mg/kg)^20^, studies of bulk sediments in continental shelf areas reveal a high content of detrital silicate materials which are known to be depleted in REE and thus act as a diluting agent^13, 33, 41^, lowering final total bulk REE concentrations at values lower than 100 mg/kg (i.e. below concentration of UCC and thus shale composition 150 mg/kg, Fig. 1f). However, total bulk REE concentrations for Amazon estuary sediments are up to 500 mg/kg^34^, and could thus be considered as a potential resource of REE.

Eventually, based on calculation proposed by Chabaux *et al*.^42^, Nd flux is equal to >1 t/year in the Garonne system, which is low, compared to other major river systems, such as the Amazon main stream and its major tributaries surrounded by many floodplains that have a maximum Nd flux equal to >1200 t/year (during high-water season), constituting 30% of the river flux to the Atlantic Ocean^39, 43^. As a consequence, one can estimate a total REE input of more than 2600 t/year for the Amazon system, divided as follows: 1940 t/year of LREE, 365 t/year of MREE and 312 t/year of HREE (Table 1). The most important fluxes are the ones observed during the high water seasons (Table 1). Eastern and Western Atlantic continental shelves should thus be considered as exploration targets with a high REE potential, especially the Amazon, Orinoco and Congo estuaries. Eventually one must also consider the onshore palaeo-sedimentary-platforms in which distal clays were deposited in a reducing marine environment with organic carbon and phosphate anomalies in the same setting^18, 44^. Such a sedimentary depositional environment should be of great interest to explore for REE especially if deep diagenesis to very low grade metamorphism conditions occurred, favoring rhabdophane (hexagonal [L-MREE]PO_4_.H_2_O) and then monazite (monoclinic [L-MREE]PO_4_) crystallization^30^. However, such sedimentary depositional environment could not directly be compared to Pacific deep sea muds^13^.

**Table 1:** Assessment of Nd, LREE, MREE and HREE inputs to the Atlantic seabed sediments supported by the calculated percent removals of Sholkovitz^24^ using data from Barroux *et al*.^43^, Dupré *et al*.^28^, Gaillardet *et al*.^29^, and Martin *et al*.^31^.

	t/year Nd	t/year LREE	t/year MREE	t/year HREE
Amazon (annual^a^)	549.2	1940.4	365.4	311.9
Amazon min (low-water stage^b^)	158.1	553.1	137.6	106.9
Amazon max (high-water stage^c^)	1215.0	4457.0	716.1	602.2
Congo max (high-water stage^c^)	431.6	1733.6	177.4	153.9
Gironde & Dordogne (annual^a^)	1.2	5.3	0.6	0.3

^a^Corresponds to average annual REE fluxes with respect to average annual discharge. ^b^Corresponds to a one-year linear extrapolation considering the minimum REE input/discharge recorded during the low-water stage. ^c^Corresponds to a linear extrapolation for one-year considering the maximum REE input/discharge recorded during the high-water stage.
